# Digital self-management interventions for osteoarthritis: a systematic scoping review of intervention characteristics, adherence and attrition

**DOI:** 10.1186/s13690-022-00854-x

**Published:** 2022-03-31

**Authors:** Rhiannon K. Patten, Alexander Tacey, Rebecca Pile, Alexandra Parker, Mary De Gori, Phong Tran, Michael J. McKenna, Rebecca Lane, Vasso Apostolopoulos, Catherine M. Said, Itamar Levinger, Mary N. Woessner

**Affiliations:** 1 Institute for Health and Sport, Victoria University, Melbourne, Australia; 2 Physiotherapy, Western Health, Melbourne, Australia; 3 Department of Orthopaedic Surgery, Western Health, Melbourne, Australia; 4 Australian Institute for Musculoskeletal Science (AIMSS), Victoria University, University of Melbourne and Western Health, Melbourne, Australia; 5 Physiotherapy, Melbourne School of Health Sciences, University of Melbourne, Melbourne, Australia

**Keywords:** Osteoarthritis, Self-management, Physical activity, Pain management, Adherence, Attrition

## Abstract

**Background:**

Osteoarthritis (OA) is a chronic, progressive condition that can be effectively managed via conservative treatments including exercise, weight management and education. Offering these treatments contemporaneously and digitally may increase adherence and engagement due to the flexibility and cost-effectiveness of digital program delivery. The objective of this review was to summarise the characteristics of current digital self-management interventions for individuals with OA and synthesise adherence and attrition outcomes.

**Methods:**

Electronic databases were searched for randomised controlled trials utilising digital self-management interventions in individuals with OA. Two reviewers independently screened the search results and extracted data relating to study characteristics, intervention characteristics, and adherence and dropout rates.

**Results:**

Eleven studies were included in this review. Intervention length ranged from 6 weeks to 9 months. All interventions were designed for individuals with OA and mostwere multi-component and were constructed around physical activity. The reporting of intervention adherence varied greatly between studies and limited the ability to form conclusions regarding the impact of intervention characteristics. However, of the seven studies that quantified adherence, six reported adherence > 70%. Seven of the included studies reported attrition rates < 20%, with contact and support from researchers not appearing to influence adherence or attrition.

**Conclusions:**

Holistic digital interventions designed for a targeted condition are a promising approach for promoting high adherence and reducing attrition. Future studies should explore how adherence of digital interventions compares to face-to-face interventions and determine potential influencers of adherence.

**Supplementary Information:**

The online version contains supplementary material available at 10.1186/s13690-022-00854-x.

## Background

Osteoarthritis (OA) is a chronic and disabling condition, characterised by joint pain and stiffness leading to loss of function and impaired quality of life [1]. OA is highly prevalent, affecting approximately 2.2 million Australians [2], with incidence estimates rising steeply with age in both males and females [3]. The knee and hip are the most commonly affected joints, ranking highly among global causes of disability and chronic pain, and contributing to a large proportion of the economic burden [4, 5]. OA is a chronic progressive condition that can be effectively managed through conservative non-surgical interventions but can often require specialist consultation and surgery [6]. The core conservative strategies recommended by evidence-based guidelines include physical activity, weight management and OA education and self-management [7–9].

Self-management is defined as the individual’s ability to manage the symptoms, treatment, physical and psychosocial consequences and lifestyle changes inherent in living with OA [10]. The core components of self-management programs recommended by national and international guidelines are OA education, physical activity and weight loss in those who are overweight or obese [6–9, 11]. Other components commonly included in self-management programs include cognitive behavioral therapy, mind-body exercise (yoga and tai-chi), aquatic exercise and use of assistive walking devices [6–9]. As pain is often the predominant symptom and a cause of significant burden, providing pain-management support is crucial [12]. Self-management programs that encompass patient education and include a cognitive behavioural component are widely recommended, and have been found to reduce pain [13, 14], enhance physical function [13] and increase self-efficacy [15].

The growing burden of OA on both the healthcare system and the individual, as well as the restrictions in face-to-face consultation due to the coronavirus disease 2019 (COVID-19) pandemic, has led to increased development and implementation of programs that can be offered digitally [16, 17]. Digital programs could be useful as they provide increased accessibility, flexibility and convenience, all at low, or no cost for the user [18]. There has been some success in reducing pain and improving function with these types of interventions for individuals with OA [13]. A recent systematic review and meta-analysis reported that digital self-management programs moderately reduced pain and improved physical function at comparable levels to face-to-face interventions [13]. However, there are often challenges that exist with digital programs that have limited practitioner support, including poor adherence and high dropout rates [19, 20].

Implementing conservative, digital self-management strategies for individuals with OA at a population level can result in substantial cost savings to the individual and the healthcare system [7]. Digital self-management interventions have the capacity to reach a large number of people, improving the dissemination of health-related education and support to individuals with OA. However, digital health interventions may also face critical barriers with engaging and retaining participants [21]. The aim of this systematic scoping review is to summarise and characterise the current digital self-management interventions in individuals with OA and synthesise adherence and attrition outcomes to these interventions.

## Methods

This scoping review adhered to the Preferred Reporting Items for Systematic reviews and Meta-Analyses extension for Scoping Reviews (PRISMA-ScR) [22] and was guided by the framework outlined by Arksey and O’Malley [23].

### Search strategy and eligibility criteria

Electronic database searches were conducted in July 2021 using EBSCOhost (CINAHL, MEDLINE, APA PsycInfo and SPORTDiscus), CENTRAL, Ovid MEDLINE and EMBASE with no date or language restrictions placed on the search. The databases selected were comprehensive and cover a broad range of disciplines. The search included a combination of Medical Subject Heading (MeSH) terms and free text keywords relating to OA, digital-based, self-management interventions. The search strategy was developed in consultation with an institutional librarian. An example search strategy is reported in Additional file 1 and was adapted for the specific requirements of each database. Reference lists of relevant review articles were also searched to identify additional eligible studies.

### Eligibility criteria

The participant, intervention, comparison, outcome and studies (PICOS) framework was used for this systematic scoping review. Randomised controlled trials that included adults (≥18 years of age) with a diagnosis of OA (self-report or by a medical practitioner), or who met the criteria for chronic hip and/or knee pain and utilised a digital-based, self-management intervention were included in this review. Digital interventions included those that were online (website) or a mobile phone application. We included interventions that utilised a self-management program, as defined by Lorig and Holman [24]. We included randomised controlled trials (RCT) with any form of control group (e.g. waitlist, treatment as usual, active controls, etc.) that reported any measure of feasibility or acceptability. Studies were excluded when participants were in an in-patient setting (hospital, nursing home or care institution), or had received surgery, and studies that utilised a supervised intervention (face-to-face or telehealth) as the primary component.

### Study selection

After the removal of duplicates, two reviewers independently screened each article by title and abstract, followed by full-text review using Covidence software (Covidence systematic review software, Veritas Health Innovation, Melbourne, Australia. Available at www.covidence.org). Discrepancies were resolved through discussion or in consultation with a third reviewer when required.

### Data charting and synthesis

A data extraction template was jointly developed a priori by two reviewers in order to determine which data to extract. The two reviewers independently piloted the data extraction template, discussed the results and iteratively updated the template. Data were extracted independently by two reviewers (R.P. and A.T.). Extracted data included study design, participant characteristics (age, BMI, sex and affected joint), main findings, intervention characteristics (duration and frequency), intervention content, mode of delivery and outcomes relating to adherence, attrition and useability. Study details, participant characteristics and intervention details were summarised. Studies were then grouped and discussed according to the content provided within their intervention, with categorisation of content being collectively agreed upon by the two reviewers. Lastly, studies reporting outcomes relating to adherence, attrition and useability were summarised.

## Results

**Fig. 1:**
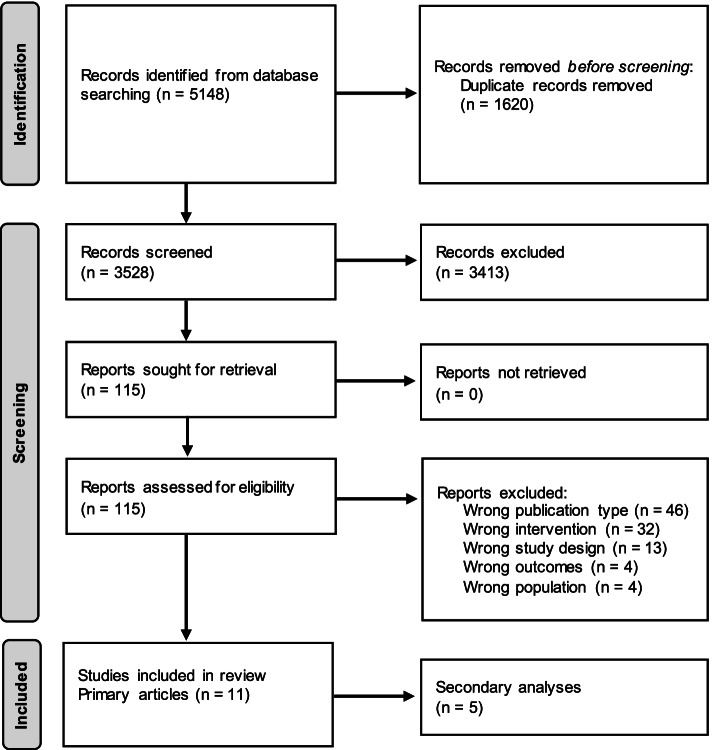
PRISMA flow diagram

A flow diagram of the study selection process is presented in Fig. 1. Overall, 5148 studies were identified in the initial database search. After 1620 duplicates were removed, 3528 were screened, from which 115 were reviewed for full-text. Of these, 16 articles met the eligibility criteria (Fig. 1). Following this, the reference lists of relevant articles were reviewed, but no additional articles were identified that fulfilled the inclusion criteria. Of the 16 eligible articles, five were secondary analyses from which data was not required for this review. Therefore, a total of 11 articles are discussed in this review.

### Summary of study details and participant characteristics

A summary of the study information, participant characteristics and main findings of the 11 included studies is reported in Table 1 and briefly described. Six studies included individuals with knee OA [25–30], one included individuals with hip OA [31] and four included individuals with either hip or knee OA [15, 32–34]. Ten studies reported a higher numbers of female participants (range, 56–100%). The mean age of participants ranged from 54 to 68 years. The sample size of the studies ranged from 40 to 427. Four studies included a follow-up at 12-months [15, 26, 31, 34], one at 9 months [28] and the remaining six studies had no follow-up [25, 27, 29, 30, 32, 33]. The control groups varied amongst included studies. Four studies provided education to their control group [27, 28, 30, 31], one provided a home-based exercise program [25], one provided usual physical therapy [34], two included a waitlist control group [15, 26] and three studies did not provide anything to their control group [29, 32, 33]. In addition to a control group, two studies also included a physical therapy group, which included individualised home exercise programs. Pain was the most commonly reported outcome used by authors to assess the effectiveness of the interventions [25, 28, 29, 32–34].

**Table 1:** Study details, participant characteristics and main findings

Study	Country	Participant characteristics Age & BMI: mean ± SD Sex: (N female/male)	Affected joint (%)	Main study findings
Alasfour & Almarwani 2020 [25]	Saudi Arabia	Age: 54.4 ± 4.3 years BMI: NR Sex: 40/0	100% knee OA	Greater improvements in pain in the intervention group compared to the control group.
Allen et al. 2018 [26]	USA	Age: 65.3 ± 11.1 years BMI: 31.4 ± 8.0 Sex: 251/99	100% knee OA	No significant differences in WOMAC scores between groups.
Allen et al. 2021 [27]	USA	Age: 60.0 ± 10.3 years BMI: 33.9 ± 7.4 Sex: 53/292	100% knee OA	Greater improvement in the total WOMAC score in the intervention group compared to control group.
Bennell et al. 2017 [28]	Australia	Age: 60.8 ± 6.5^a^ years BMI: 32.0 ± 13.9^a^ Sex: 83/65	100% chronic knee pain suggestive of OA	Significant improvement in pain and physical function in the intervention group compared to the control group.
Bennell et al. 2018 [31]	Australia	Age: 61.2 ± 7.2^a^ years BMI: 29.2 ± 13.1^a^ Sex: 82/62	100% hip OA	No significant differences between groups in pain or physical function.
Bossen et al. 2013 [15]	The Netherlands	Age: 62.0 ± 5.7 years BMI: 27.6 ± 4.5 Sex: 129/70	64% knee OA, 21% hip OA and 15% had both	Significant improvements in physical function in the intervention group compared to the control group.
Gohir et al. 2021 [29]	UK	Age: 66.7 ± 9.2 years BMI: 31.9 ± 5.9^a^ Sex: 71/34	100% knee OA	Significant improvements in pain and physical function in the intervention group compared to the control group.
Kloek et al. 2018 [34]	Netherlands	Age: 63.8 ± 4.2^a^ years BMI: 27.8 ± 4.2^a^ Sex: 141/67	67% knee OA, 18% hip OA and 15% had both	Both groups significantly improved pain, quality of life and self-efficacy. No significant differences between the groups.
Nelligan et al. 2021 [30]	Australia	Age: 60.0 ± 8.0 years BMI: 31.1 (26.6–34.9)^a,b^ Sex: 109/97	100% knee OA	Significant improvements in pain and physical function in the intervention group compared to the control group.
Pelle et al. 2020 [32]	Netherlands	Age: 62.1 ± 7.3 years BMI: 27.8 ± 5.1^a^ Sex: 306/119	73% knee OA and 27% hip OA	No significant difference in health care utilisation between the two groups. Significant improvements in pain, symptoms and activities of daily living in the intervention group.
Rini et al. 2015 [33]	USA	Age: 67.6 ± 9.5 years BMI: NR Sex: 91/22	35% knee OA, 12% hip OA and 52% had both	Significant improvements in pain among women who received intervention compared to the control group. Both men and women increased self-efficacy post-intervention compared to the control group.

*Abbreviations*: *BMI* body mass index, *OA* osteoarthritis, *PT* physical therapy, *UK* United Kingdom, *USA* United States of America, *WOMAC* Western Ontario and McMaster Universities Osteoarthritis Index

^a^Intervention group only

^b^Median and interquartile range

### Intervention summary

The intervention details are reported in Table 2. All interventions were designed specifically for individuals with OA. The length of the interventions ranged from 6 weeks to 9 months. Four interventions were less than 10 weeks [15, 25, 29, 33], three were between 10 and 20 weeks [26, 28, 34] and four were longer than 20 weeks [27, 30–32]. Eight studies utilised a website to deliver their intervention [15, 26–28, 30, 31, 33, 34] and the remaining three studies used a smartphone application [25, 29, 32].

**Table 2:** Intervention details

Study & delivery	Duration & frequency	Physical activity	OA education	Nutrition	Pain management	CBT	Other (reminders, equipment, etc.)
Alasfour & Almarwani 2020 [25] App-based	Duration: 6 weeks Frequency: NR	Strengthening exercises for lower-limb muscles	X	X	X	X	• Alerts and monitoring system. • Resistance band
Allen et al. 2018 [26] Internet-based	Duration: 16 weeks Frequency: 3 times per week	Tailored strengthening, stretching and aerobic exercise with progressions	X	X	Pain monitoring	X	• Automated reminders • Progress tracking • Ankle weights and resistance bands
Allen et al. 2021 [27] Internet-based	Duration: 9 months Frequency: 3 times per week	Tailored strengthening, stretching and aerobic exercise with progressions	X	X	Pain monitoring	X	• Automated reminders • Tracking progress • Ankle weights and resistance bands
Bennell et al. 2017 [28] Internet-based	Duration: 12 weeks Frequency: 1 PCST module and 3 exercise sessions per week	Individualised lower-limb-strengthening exercise program	Weeks 1–8: Education material on a range of topics^a^	Education about healthy eating	Education regarding pain management	Weeks 1–8: PCST^b^	• Resistance bands and ankle weights • Email reminders • Optional pedometer
Bennell et al. 2018 [31] Internet-based	Duration: 24 weeks Frequency: 1 PCST module and 3 exercise sessions per week	Weeks 8–24: Individualised lower-limb-strengthening and flexibility exercises	Weeks 1–8: Education material on a range of topics^a^	Education about healthy eating	Education regarding pain management	Weeks 1–8: PCST^b^	• Email reminders
Bossen et al. 2013 [15] Internet-based	Duration: 9 weeks Frequency: 1 module per week	Self-paced, graded, self-selected physical activity program	Education about OA	X	X	X	• Web-based messages and emails • Goal settings
Gohir et al. 2021 [22] App-based	Duration: 6 weeks Frequency: daily exercises	Leg strengthening, core stability and balance	Education about OA, treatments, managing symptoms, behaviour change and healthy lifestyle	X	X	X	• Daily emails or notifications • Quizzes
Kloek et al. 2018 [34] Internet-based	Duration: 12 weeks Frequency:	Self-selected aerobic exercise and 2 strength exercises progressed gradually	Weekly videos about OA etiology, medication, and social influences on pain	Information regarding weight management	Education regarding pain management	X	•Weekly automatic emails •Tailored feedback
Nelligan 2021 [30] Internet-based	Duration: 24 weeks Frequency: 3 times per week	Lower limb strengthening exercise program	Education about living with knee OA and treatments	X	Education about managing exercise pain	X	• Regular automated messages and prompts •Logbooks
Pelle et al. 2020 [32] App-based	Duration: 26 weeks Frequency: daily exercises	Exercise library containing 10 exercises	OA education and treatment, generic lifestyle advice, physical activity and vitality	Information and goal setting regarding nutrition for weight management	X	Goal setting	• Tailored goals • Daily notification /reminders •Rewards
Rini et al. 2015 [33] Internet-based	Duration: 8 weeks Frequency: 1 PCST module per week	X	X	X	Education regarding pain management	PCST^b^	•Automatic reminders • Earn badges • COACHtrack to self-monitor • COACHchat for social support

*CBT* cognitive behavioural therapy, *NR* not reported, *PCST* pain-coping skills training

X = not included in intervention

^a^Education material covering exercise and physical activity, pain management, emotions, healthy eating, complementary therapies, and medications from www.arthritisaustralia.com.au

^b^PCST included progressive relaxation, activity-rest cycling, scheduling pleasant activities, changing negative thoughts, pleasant imagery and distraction techniques, and problem solving

### Summary of intervention content

#### Physical activity

A broad range of physical activity prescriptions were utilised in the included studies. Ten of the 11 studies included a physical activity component, eight of which focused their exercise prescription around lower-limb strengthening exercises [25–29, 31, 32, 35], one included both strength exercises and aerobic exercise [25] and one included only aerobic exercise [30]. Two of the studies that primarily focused on strengthening exercise also provided aerobic exercise recommendations [26, 27]. Four studies provided individualised exercise prescription [26–28, 31]. Six of the studies included exercise progressions for the lower limb strength exercises [26–29, 31, 34], one study gave participants the ability to alter the intensity of their aerobic exercise [15], one study periodically increased the amount of exercises completed per week [25] and the remaining two studies did not report progressions [30, 32]. Six studies requested participants complete their recommended exercises three times per week [26–28, 30, 31, 34], two studies recommended daily exercises [29, 32], one study asked participants to complete one module per week [15] and the remaining study did not report the frequency of exercise [25]. No studies reported prescribed exercise intensity.

#### Education

A diversity of educational content were delivered. Seven of the studies included a component of OA disease-specific education [15, 28–32, 34]. The most common topics covered were OA treatments, and the benefits of behavioural change and lifestyle modifications for improving the symptoms of OA. Other topics included OA aetiology, medications, vitality, nutrition and alternative therapies.

#### Weight management/healthy eating

Only four studies included a component addressing healthy eating/weight management [28, 31, 32, 34]. One of these provided nutritional information for weight management and included goal setting on nutrition to promote weight management [32]. Two studies focused on education regarding healthy eating [28, 31] and the fourth study provided education on weight management [34].

#### Pain management and cognitive behavioural therapy/behaviour change techniques

Seven studies included a component of pain management [26–28, 30, 31, 33, 34]. Two of these studies only provided pain monitoring [26, 27], two provided information regarding pain management [30, 34] and the remaining three studies used a pain-coping skills training (PCST [PainCOACH]) program, which uses cognitive behavioural therapy principles to manage or reduce pain [28, 31, 33]. The PainCOACH program included eight modules, one completed per week, each providing interactive training in a cognitive or behavioural pain coping skill. The modules covered progressive muscle relaxation, activity-rest cycling, scheduling pleasant activities, changing negative thoughts, pleasant imagery and distraction techniques and problem solving.

One study also had a strong focus on goal setting with tailored goals [32], with the central feature of the application used being a library of predefined “tiny habit” goals and triggers to a healthier lifestyle.

#### Social/peer support

Only one of the included studies had a component of social or peer support [33]. Participants were able to post their own experiences and read about the experience of others.

#### Contact with study personnel

Four of the studies provided a phone number or an email address for participants to contact the research team if they needed assistance [26, 27, 29, 30]. Five studies either did not provide or did not report whether participants were given details to contact the research team [15, 25, 31–33]. Two studies provided either teleconferencing [28] or face-to-face consults with participants [34]. Every study utilised automated reminders sent via email or text message, or from within the website, and ranged from daily to monthly reminders.

### Adherence, attrition, usability and satisfaction

#### Adherence to intervention

Adherence outcomes are reported in Table 3. Of the 10 studies utilising a home exercise program, five reported adherence rates. Four studies reported adherence levels ranging from 68.0 [28] to 87.9% [29] and one used the exercise adherence rating scale, reporting a score of 15.4/24 [30]. The study reporting the greatest adherence to the exercise program (87.9%) utilised an app-based program for 6 weeks [29]. Another 6 week app-based intervention reported an average adherence of 83.4% [25]. The lowest mean adherence to a home exercise program of 68% was for an intervention of 12 weeks duration [28]. The two studies with the highest reported levels of exercise adherence were app-based studies [25, 29], whereas the two studies with the lowest reported adherence were online interventions [28, 31].

**Table 3:** Feasibility outcomes

Study	Group size (total N)	Adherence	Dropout rates post-intervention	Dropout rates at follow up	Satisfaction
Alasfour & Almarwani 2020 [25]	I: 20 C: 20 (40)	• An average of 85% of exercise sessions were completed	10%	X	X
Allen et al. 2018 [26]	I: 142 C: 68 (350)	• 80% logged on to program at least once • Mean of 21 logins per participant	25%	27%	X
Allen et al. 2021 [27]	I: 230 C: 115 (345)	• 72% logged on to program at least once • Median of 2 logins per participant (median of 11 for those that logged in at least once)	29%	X	X
Bennell et al. 2017 [28]	I: 74 C: 74 (148)	• 68% of home exercise sessions completed • 78% accessed education material • 64% of PCST practices completed • Average of 6.4 of 8 PCST modules were completed	5%	11%	• Education = 1.8/5^a^ • PCST = 2/5^a^ • Physiotherapy = 1/5^a^
Bennell et al. 2018 [31]	I: 73 C: 71 (144)	• 72% of home exercise sessions completed • 74% accessed educational material • Average of 6.6 of 8 PCST modules were completed	9%	12%	17 questions on the usability of PCST. See paper for details.
Bossen et al. 2013 [15]	I: 100 C: 99 (199)	• 94% logged on at least once • 62% of education modules completed • 46% adherent to the intervention (6/9 modules completed) • 19% completed all modules	16%	24%	SUS = 73/100^a^
Gohir et al. 2021 [29]	I: 48 C: 57 (105)	• An average of 88% of exercise sessions were completed	28%	X	X
Kloek et al. 2018 [34]	I: 109 C: 99 (208)	• 81% of participants adhered to the program (completed at least 8 of the 12 modules)	18%	39%	SUS = 73/100^a^
Nelligan et al. 2021 [30]	I: 103 C: 103 (206)	• 97% logged on at least once • 39% accessed the website in final 4 weeks • Mean of 6 logins per participant • 73% reply rate to text messages	13%	X	• Treatment satisfaction = 5.6/7^a^ • Usefulness of website = 5.3/7^a^ • Usefulness of text messages = 5.3/7^a^
Pelle et al. 2020 [32]	I: 214 C: 213 (427)	• 80% opened the app at least once • 70% adherent to intervention (chose at least one goal) • 53% achieved at least one goal • 26% still used intervention at the end of the study	39%	X	SUS = 65/100^a^
Rini et al. 2015 [33]	I: 58 C: 55 (113)	•91% completed all PCST modules	2%	X	X

All reported data is for intervention group at the main assessment time point

X = outcome not reported

*Abbreviations*: *C* control, *I* intervention, *PCST* pain-coping skills training, *SUS* system usability scale

^a^A higher score indicates greater satisfaction

Five studies reported average module completion rates, with rates ranging between 62 and 91% [15, 28, 31, 33, 34]. Three of these studies reported module completion rates for the pain-coping skills training (PainCOACH) program [28, 31, 33]. Two studies reported an average of 6.4 [28] and 6.8 [31] out of 8 modules were completed by participants whilst the third study reported that 91% [33] of participants completed all modules. The remaining two studies examined module completion rates relating to weekly exercise assignments [15, 34]. One study reported that 46% of participants reached the set adherence threshold of 6 out of 9 modules completed, reporting an average of 5.6 of the 9 modules were completed by participants [15]. The second study reported that 81.1% of participants completed at least 8 of the 12 weekly modules [34]. Both of these studies used a self-selected aerobic exercise, however, the latter also included strength exercises on 3 days of the week.

#### Dropouts

Dropout rates varied from 1.7% [33] to 39.3% [32] (Table 3). Of these, one study reported a drop out rate > 30% [32], three studies reported a dropout rate of 20–30% [26, 27, 29], four studies reported a drop out rate between 10 and 20% [15, 25, 30, 34], and three studies reported dropout rates < 10% [28, 31, 33]. The lowest dropout rates were reported among interventions of shorter interventions with the lowest dropout rates (1.7%) occurring in an 8-week intervention [33]. Similarly, there was a dropout rate of 5% after a 6-week intervention [25]. One exception was a 6-week intervention, which recorded the second largest dropout rate at 28%, however, this study lost approximately 20% of participants due to COVID-19 lockdowns preventing post-intervention testing [29]. The largest dropout rate (39.3%) was reported in a 26-week intervention [32]. However, other 24-week interventions recorded much lower dropout rates ranging from 9.6 to 12.6%. Of the three 12-week interventions, dropout rates ranged from 5.4 to 19.7%. Dropout rates at follow up were only available for six studies and varied between 11 and 39% [15, 26–28, 31, 34].

#### Satisfaction and usability of the intervention

Six of the 11 studies reported satisfaction and usability outcomes. The system usability scale (SUS) was utilised by three studies [15, 32, 34] and a numerical rating scales (NRS) of treatment satisfaction was used by the other three studies [28, 30, 31]. The SUS scores ranged from 65 to 73/100 and are considered average scores [36]. A higher score suggests the intervention was more usable. The study with the lowest SUS score was also the study with the highest dropout rate and one of the longest intervention durations [32]. Treatment satisfaction assessed using a NRS were measured using several different scales and the results are thus difficult to interpret.

## Discussion

Despite guidelines for OA management clearly indicating key topics of importance, the online programs varied widely, not only in intervention content, but also in their duration, type of OA, and reporting of adherence measures. All interventions were, however, multi-component, included alerts and monitoring, and all but one intervention included physical activity. It appears that, irrespective of the content of the self-management interventions, there was a relatively high adherence and low attrition to the online programs, with the majority of studies reporting a dropout rate of less than 20%. It was, however, noted that interventions of longer duration tended to have higher dropout rates. Therefore, future studies should address barriers to long-term adherence to improve the impact of conservative, cost-effective therapy for individuals with OA.

Overall, the content covered in the online programs was quite diverse. Physical activity was the most common topic covered in self-management interventions, included in all but one program, which is in line with current evidence-based recommendations [6–9]. Despite weight management also being considered a core component of OA management, surprisingly, less than 40% of studies included this topic [7, 8]. It is not clear why weight management was not covered in all programs, particularly since all studies reported a mean BMI > 25 kg/m^2^_,_ which indicates that most participants were overweight or obese, and likely increasing the progression of osteoarthritis [37]. The majority of the interventions also focused specifically on either knee or hip OA, with only four designed for both. Although the management of OA in these two joints is very similar, there are slight differences, particularly in regards to exercise prescription [7, 11]. The duration of the interventions also varied greatly making it difficult to determine factors that may promote long-term adherence. Furthermore, only two studies reported adherence to the program between the end of the intervention and follow-up, both reporting a further decrease of ~ 20% adherence to the program. It is important that future studies measure long-term adherence to the intervention.

The various measures of program use and adherence, and the lack of reporting in some instances make it difficult to form definitive conclusions regarding factors that may have contributed to higher adherence. Overall we found that, unlike other digital interventions, which commonly report adherence rates of only around 50% [38, 39], studies included in this review typically reported adherence rates of 70% or greater. Three studies that reported high adherence utilised pain-coping skills training. The pain-coping skills training uses cognitive behavioural therapy and behaviour change principles to help individuals manage OA pain [40]. Cognitive behavioural therapy has been found to be beneficial for a range of health conditions including chronic pain [41], nonspecific back pain [42], mental disorders [43] and fibromyalgia [44]. The Osteoarthritis Research Society International guidelines also recommend cognitive behavioural therapy when combined with a component of exercise for individuals with knee OA [8]. Furthermore, a secondary analysis of one of these studies indicated that participants reported that they were better able to cope with the pain due to the pain-coping techniques and training utilised [45]. Autonomous motivation is an important predictor of health behaviour change and maintenance [46]. Another potential reason why adherence rates were relatively high in these studies is the self-selection of participants. Participants were recruited via advertisements and letters of invitations introducing self-selection bias, suggesting that those willing to participate in an online intervention are more willing to make a lifestyle change and therefore more likely to adhere to the intervention compared to those recruited from an inpatient setting or hospital waiting list [47].

Despite high dropout rates being a common concern among digital interventions [21], studies included in this review had relatively low dropout rates of between 5 and 39%, and only four of the included studies had dropout rates higher than the generally accepted rate of 20% [26, 27, 29, 32]. However, one of these studies was impacted by COVID-19 lockdowns, preventing approximately 20% of participants from completing post-intervention testing, inflating the dropout rate [29]. The other studies with higher dropout rates had relatively long interventions of 16 weeks or greater, suggesting the prolonged study length may have contributed to higher dropout rates. Notably, one 26 week intervention with high attrition rates also reported the lowest SUS score [32]. In contrast, two studies with interventions of 12 and 24 weeks reported low dropout rates [28, 31]. Although speculative, the fact that both studies were multidisciplinary, comprehensive interventions that utilised a combination of physical activity, education, pain management and cognitive behavioural therapy could have contributed to lower attrition rates.

The high adherence and low dropout rates reported may also be due to increased public understanding of the importance of self-management in chronic disease management, providing individuals with the tools to effectively manage their illness and improve health outcomes [48]. Adding to this, one of the most successful and well-known OA self-management programs, the Arthritis Self-Management Program, has been adopted worldwide [49, 50] and has been used as a foundation for most existing OA self-management interventions. Although these interventions are typically not delivered digitally, a recent meta-analysis exploring the impact of digital self-management interventions for people with OA demonstrated that such programs can result in a significant reduction in pain compared to a control group [13]. Although not included as a main outcome of this review, it was noted that a large number of studies included in this review reported significant improvements in their main outcome, most commonly pain, between the intervention and control groups.

We also must consider the impact of the increased accessibility of online interventions in that they allow people to receive treatment at any time and location [51]. Digital-based interventions are also more cost-effective compared to face-to-face interventions and reduce traditional barriers to treatment such as time scheduling, missing work and travel [52]. A challenge with digital interventions is that many are typically delivered only in one language and often require a certain level of literacy. Given the cultural diversity in most countries, the potential reach of these online self-management interventions will be limited if they are not developed to accommodate for individuals from linguistically diverse backgrounds. Furthermore, most self-management interventions are primarily available through participation in clinical trial evaluations. Hence, making these interventions available to the wider population could be beneficial for reducing the burden on individuals with OA and on the healthcare system.

One limitation of this review is that we were unable to differentiate the characteristics, and adherence and attrition rates between hip and knee OA due to the small number of included studies, only one of which focused on hip OA. Therefore, future research is required to determine whether knee and hip OA have different needs, in order to encourage adherence and reduce attrition to an intervention. Furthermore, some studies did not adequately report adherence or program usage, which made it difficult to determine the impact of the interventions on these outcomes. It is important for future studies to assess and report on intervention adherence and program usage in order to determine which characteristics are successful for improving these outcomes. In addition, very few studies stated whether their intervention used was designed following a theoretical framework. This information is crucial for understanding behaviour change and adherence. The application of theory is advocated as an integral step in intervention design and evaluation [53]. Although it is likely that the included studies were grounded in appropriate theories, many made no mention of such. Future studies should specifically state which theories were targeted in the development of the digital self-management interventions for individuals with OA in order to understand whether these may have impacted adherence. Lastly, studies analysing satisfaction and usability used various outcome measures, making it difficult to compare the results and form conclusions. Future studies should report participant satisfaction and use validated outcomes measures such as the SUS to determine the level of participant satisfaction and inform future interventions.

## Conclusion

In conclusion, the majority of self-management interventions for individuals with OA have been successful in promoting adherence and reducing attrition to these interventions. Given the flexibility, availability and accessibility of these programs, whilst maintaining participant adherence, digital self-management interventions could offer an opportunity for individuals with OA to self-manage symptoms and reduce their need for more invasive treatments. Future digital self-management interventions should be multi-component and include physical activity and other key aspects of osteoarthritis management such as weight management, education and cognitive-behavioural approaches to pain management to increase engagement with the intervention. In addition, future studies should consider strategies to promote long-term adherence and determine whether long-term adherence to lifestyle behaviours results in ongoing reductions in pain.

## Supplementary Information

**Additional file 1.** Example search strategy used for EBSCO.

## Data Availability

Not applicable, all data are available within the manuscript.

## Abbreviations

BMI: Body mass index
CBT: Cognitive behavioural therapy
COVID-19: Coronavirus disease 2019 (COVID-19)
OA: Osteoarthritis
PCST: Pain-coping skills training
PT: Physical therapy
RCT: Randomised controlled trials
SUS: System usability scale
WOMAC: Western Ontario and McMaster Universities Osteoarthritis Index
