# Supplementary material for: Digital self-management interventions for osteoarthritis: a systematic scoping review of intervention characteristics, adherence and attrition
Source: Arch Public Health. 2022 Mar 31;80:103. doi: 10.1186/s13690-022-00854-x (PMC8968262; doi:10.1186/s13690-022-00854-x)
Supplement: Supplementary file 1 — Additional file 1. Example search strategy used for EBSCO. [file 13690_2022_854_MOESM1_ESM.docx]

**Supplementary material**

Additional file 1: Example search strategy used for EBSCO

Osteoarthr* or arthr* or hip pain or knee pain or joint pain

AND

Self-management or self management or physical activity or exercise or education or weight management or weight loss or cognitive behavioural therapy or cognitive behavioral therapy or mental health or intervention or pain management or social support or nutrition or diet or self care or self-care or program or programme

AND

Online or digital or ehealth or e-health or e health or telehealth or mhealth or m-health or m health or application or apps or app or mobile or smartphone or smart-phone or telerehab* or telemed* or telehealth or telecare or teletherap* computer or website or web-based or web based or internet or internet-based or technolog* or telephysio* or telephone

AND

Randomised controlled trial or randomized controlled trial or randomised control trial or randomized control trial or randomly or randomised or randomized or clinical or randomlySupplementary Table 2. Study details, participant characteristics and main findings.

| Study | Country | Participant characteristics  Age & BMI: mean ± SD  Sex: (N female/male) | Affected joint (%) | Main study findings |
| --- | --- | --- | --- | --- |
| Alasfour & Almarwani 2020^25^ | Saudi Arabia | Age: 54.4 ± 4.3 years  BMI: NR  Sex: 40/0 | 100% knee OA | Greater improvements in pain in the intervention group compared to the control group. |
| Allen et al. 2018^26^ | USA | Age: 65.3 ± 11.1 years  BMI: 31.4 ± 8.0  Sex: 251/99 | 100% knee OA | No significant differences in WOMAC scores between groups. |
| Allen et al. 2021^27^ | USA | Age: 60.0 ± 10.3 years  BMI: 33.9 ± 7.4  Sex: 53/292 | 100% knee OA | Greater improvement in the total WOMAC score in the intervention group compared to control group. |
| Bennell et al. 2017^28^ | Australia | Age: 60.8 ± 6.5^a^ years  BMI: 32.0 ± 13.9^a^  Sex: 83/65 | 100% chronic knee pain suggestive of OA | Significant improvement in pain and physical function in the intervention group compared to the control group. |
| Bennell et al. 2018^31^ | Australia | Age: 61.2 ± 7.2^a^ years  BMI: 29.2 ± 13.1^a^  Sex: 82/62 | 100% hip OA | No significant differences between groups in pain or physical function. |
| Bossen et al. 2013^15^ | The Netherlands | Age: 62.0 ± 5.7 years  BMI: 27.6 ± 4.5  Sex: 129/70 | 64% knee OA, 21% hip OA and 15% had both | Significant improvements in physical function in the intervention group compared to the control group. |
| Gohir et al. 2021^29^ | UK | Age: 66.7 ± 9.2 years  BMI: 31.9 ± 5.9^a^  Sex: 71/34 | 100% knee OA | Significant improvements in pain and physical function in the intervention group compared to the control group. |
| Kloek et al. 2018^34^ | Netherlands | Age: 63.8 ± 4.2^a^ years  BMI: 27.8 ± 4.2^a^  Sex: 141/67 | 67% knee OA, 18% hip OA and 15% had both | Both groups significantly improved pain, quality of life and self-efficacy.  No significant differences between the groups. |
| Nelligan et al. 2021^30^ | Australia | Age: 60.0 ± 8.0 years  BMI: 31.1 (26.6-34.9)^a,b^  Sex: 109/97 | 100% knee OA | Significant improvements in pain and physical function in the intervention group compared to the control group. |
| Pelle et al. 2020^32^ | Netherlands | Age: 62.1 ± 7.3 years  BMI: 27.8 ± 5.1^a^  Sex: 306/119 | 73% knee OA and 27% hip OA | No significant difference in health care utilisation between the two groups. Significant improvements in pain, symptoms and activities of daily living in the intervention group. |
| Rini et al. 2015^33^ | USA | Age: 67.6 ± 9.5 years  BMI: NR  Sex: 91/22 | 35% knee OA, 12% hip OA and 52% had both | Significant improvements in pain among women who received intervention compared to the control group.  Both men and women increased self-efficacy post-intervention compared to the control group. |

Abbreviations. BMI: body mass index, OA: osteoarthritis, PT: physical therapy, UK: United Kingdom, USA: United States of America, WOMAC: Western Ontario and McMaster Universities Osteoarthritis Index

^a^Intervention group only

^b^Median and interquartile range
